# Kapandji-assisted Closed Reduction and Percutaneous K-wire Fixation for Proximal Humerus Fractures in Adolescents: A Technical Note

**DOI:** 10.1055/s-0044-1787549

**Published:** 2024-08-01

**Authors:** Florencia Turazza, Heloisa Zimmermann Faggion, Julio Javier Masquijo

**Affiliations:** 1 Departamento de Ortopedia Infantil, Sanatorio Allende, Córdoba, Argentina; 2 Hospital do Trabalhador, Curitiba, PR, Brasil

**Keywords:** adolescents, closed reduction, humeral fractures, Kirschner wire

## Abstract

Proximal humerus fractures account for approximately 3 to 5% of all pediatric-adolescent fractures, with a higher incidence observed in older children, particularly between the ages of 10 and 15 years. Non-displaced or minimally displaced fractures can often be treated conservatively. However, the management of displaced or unstable proximal humerus fractures in adolescents may involve surgical intervention, with closed reduction and percutaneous pinning (CRPP) being commonly employed techniques. Closed reduction and percutaneous pinning is not without its challenges and potential difficulties. This article aims to describe a technique that can facilitate CRPP and minimize complications associated with this surgical approach.

## Introduction

Proximal humerus fractures account for approximately 3 to 5% of all pediatric fractures, with a higher incidence observed in older children, particularly between the ages of 10 and 15 years. 1 Male children tend to be more affected than females. These fractures commonly result from falls, sports-related injuries, or direct trauma to the shoulder region.

The management of proximal humerus fractures in adolescents depends on various factors, including fracture characteristics, age, and skeletal maturity. Non-displaced or minimally displaced fractures can often be treated conservatively with immobilization using slings or braces. However, displaced or unstable fractures in patients with a limited remodeling potential may require surgical intervention. Closed reduction and percutaneous pinning (CRPP) are commonly employed surgical techniques for the management of displaced or unstable proximal humerus fractures in adolescents. 2 3 4 While these procedures can be effective in achieving anatomical alignment and promoting fracture healing, they are not without challenges and potential difficulties. This article aims to describe a technique that can facilitate CRPP and minimize complications associated with this surgical approach.

## Technical Description

**Fig. 1:**
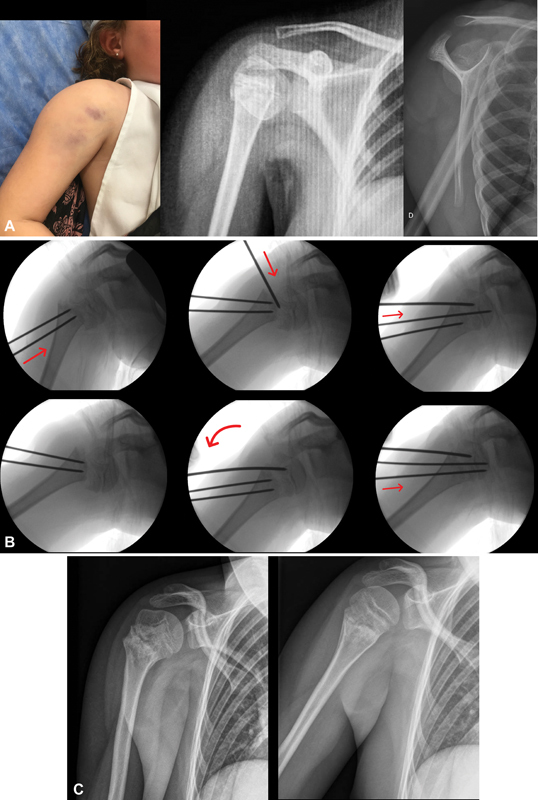
A) Clinical image and radiographs at initial presentation. B) Fluoroscopic images during closed reduction and percutaneous pinning. C) Anteroposterior and lateral radiographs at 3 months follow-up.

**Fig. 2:**
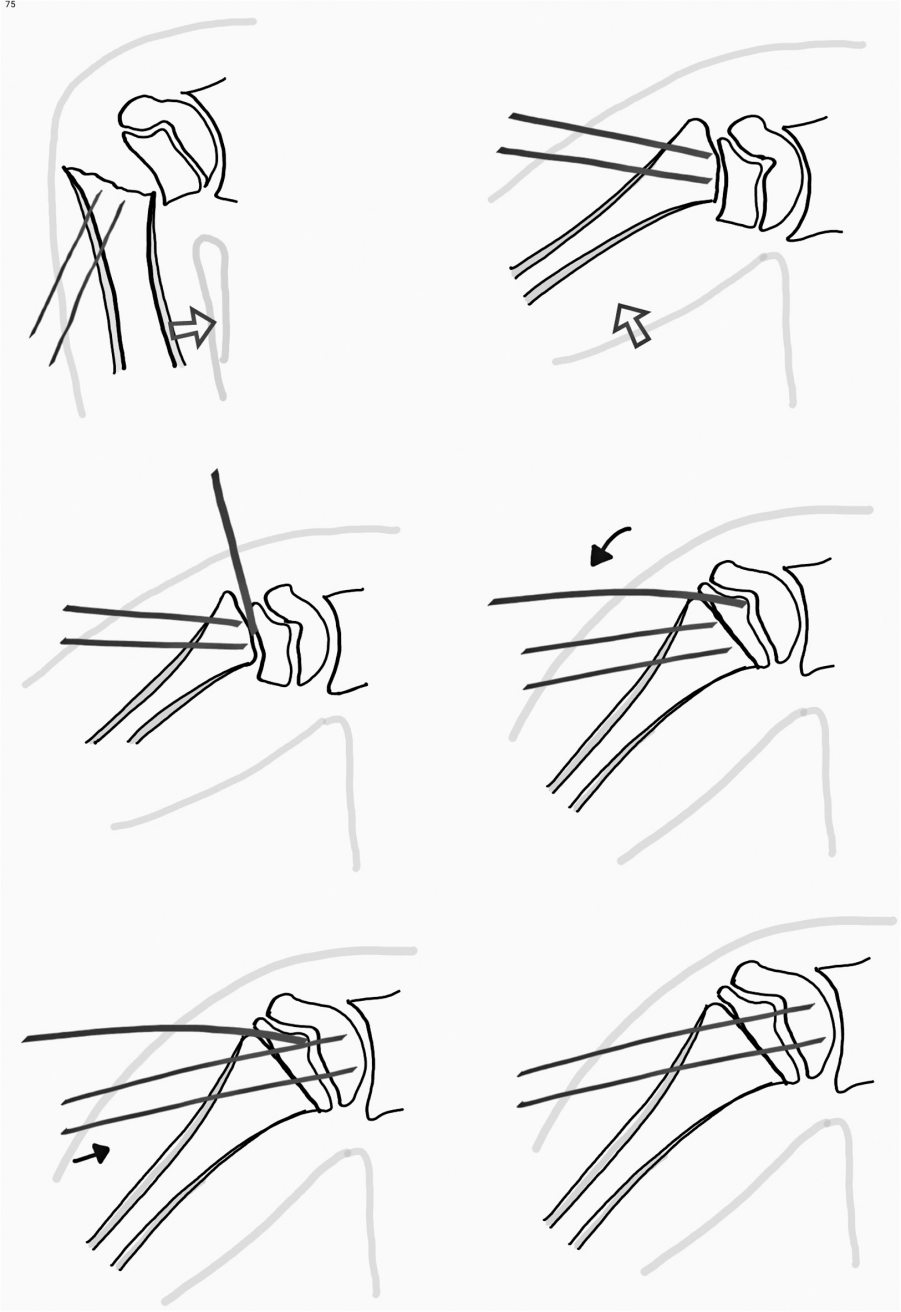
Diagram showing the Kapandji-assisted closed reduction and percutaneous K-wire fixation for proximal humerus fractures.

The indications for surgical management of proximal humerus fractures are described in Table 1 . Reduction is considered for fractures that are displaced by more than 33% (Neer III or IV) or fractures exhibiting an angulation of over 40 degrees in patients who are within 2 years of reaching skeletal maturity (boys aged ≥ 14 years and girls aged ≥ 12 years). 5 The patient is positioned in the supine or modified beach chair position to allow orthogonal imaging ( Figs. 1 and 2 ). Subsequently, the entire upper extremity is draped. With the arm in adduction, 2 2.0-mm Kirschner wires are percutaneously inserted and advanced to the fracture site. In this position, it is easier to introduce pins than with the arm in abduction and rotation. It is crucial to avoid the axillary nerve during percutaneous placement of the pins. The nerve is located within 6 cm of the anterolateral aspect of the acromion, and the pins are placed distal to this site. Once the position of the pins has been confirmed radiographically, a closed reduction of the fracture is performed with traction, abduction, and rotation. If the reduction is deemed inadequate, a 2.5-mm Steinmann pin is used to facilitate the reduction (Kapandji technique). Occasionally, the periosteum or the long head of the biceps may interfere at the fracture site, requiring an open reduction through a deltopectoral approach. Once reduction is achieved, the pins are advanced, and the Steinmann pin is removed. It is important to avoid penetrating the articular surface with the Kirschner wires. Subsequently, tests are performed with continuous fluoroscopy for maximum internal and external rotation to assess stability and the final position of the pins. If signs of fracture instability are observed, a third 2.0-mm crossed pin is inserted. The wires are then cut, bent, and kept outside the skin, thus allowing for their removal in the office.

**Table 1:** Indications for surgical management of proximal humerus fractures in adolescents.

Indications
Open fractures
Fractures associated with neurovascular compromise
Skin tenting or risk of impending open fracture
Older children approaching skeletal maturity with displaced and angulated fractures
Polytrauma requiring early weight-bearing of the affected upper extremity
Ipsilateral extremity fractures (floating elbow)

Postoperatively, the affected limb is placed in a sling with a cushion to maintain a certain degree of abduction. The fracture is followed biweekly to assess healing, and the sling and pins are removed at 4 weeks in the office. Passive and progressive active shoulder range-of-motion exercises, including forward elevation and external rotation with the arm at the side, as well as pendulum exercises, are initiated following pin removal.

## Discussion

The proximal humeral physis exhibits remarkable potential for remodeling, enabling significant tolerance for deformities in skeletally immature children. However, surgical intervention may be necessary in certain situations. This article presents a straightforward and effective technique that facilitates CRPP, minimizing the operative time and complications associated with this surgical approach ( Table 2 ).

**Table 2:** Recommendations on how to avoid common pitfalls in closed reduction and percutaneous pinning of proximal humerus fractures in adolescents

Pitfalls	How to avoid them
Axillary nerve injury	The nerve is located within 6 cm of the anterolateral aspect of the acromion, and the pins should be placed distal to this site
Articular surface penetration	Perform continuous fluoroscopy to assess the final position of the pins in all views
Loss of fixation	Perform continuous fluoroscopy for maximum internal and external rotation to assess stability
Skin necrosis	Evaluate the need of relaxing skin incisions at the site of pin insertion
Pin-site infection	Prophylactic antibiotics, pin care, and close observation

The utilization of CRPP encounters two main challenges: 1) reduction, as controlling the proximal fragment becomes quite challenging due to muscle forces, and 2) fixation, since achieving the required oblique entry angle for pins to traverse the fracture may pose difficulties.

The closed reduction maneuver involves the application of axial traction, abduction, and external rotation to the arm. However, inserting K-wires in this position presents significant challenges due to the oblique entry angle required to traverse the fracture in hard cortical bone. To overcome this difficulty, the use of a drill guide has been proposed to ensure a more secure wire anchoring. Nonetheless, it is common for the pins to skive when attempting a distal-to-proximal trajectory. 6 Based on our experience, we have found that percutaneously placing 2 2.0-mm Kirschner wires until reaching the fracture with the arm in adduction is easier than introducing the pins in the reduction position. Once the appropriate position of the pins is confirmed using fluoroscopy, a closed reduction of the fracture is performed, and the pins are advanced into the epiphysis. Traditionally, if this maneuver does not achieve satisfactory reduction, one would perform an open reduction with a deltopectoral approach, which is not without risks. In our proposed technique, in situations in which the reduction is inadequate with closed maneuvers, a 2.5-mm Steinmann pin is employed to assist with the reduction using a Kapanji-type maneuver. This technique can aid in achieving proper alignment of the fracture avoiding an open reduction. Recently, Goldstein et al. 6 proposed the use of a blunt hemostat for similar purposes. This tool provides safe access to the fracture site, leverages the cortex of the distal fragment, and translates it to align with the proximal humerus.

## Final Considerations

In summary, the majority of proximal humerus fractures in the pediatric-adolescent population can be managed without surgery owing to its remarkable remodeling potential. However, in cases in which angulation and displacement exceed the acceptable limits, our CRPP technique can provide a reliable approach for achieving satisfactory reduction and stable fixation. This method offers a reproducible means to address fractures that require surgical intervention.

## References

[1] Peterson C A Peterson H A Analysis of the incidence of injuries to the epiphyseal growth plate J Trauma 1972 12 04 275 281 5018408 10.1097/00005373-197204000-00002

[2] Dobbs M B Luhmann S L Gordon J E Strecker W B Schoenecker P L Severely displaced proximal humeral epiphyseal fractures J Pediatr Orthop 2003 23 02 208 215 12604953

[3] Pahlavan S Baldwin K D Pandya N K Namdari S Hosalkar H Proximal humerus fractures in the pediatric population: a systematic review J Child Orthop 2011 5 03 187 194 21779308 10.1007/s11832-011-0328-4 PMC3100455

[4] Swarup I Hughes M S Bram J T Horn B D Ganley T J Percutaneous pinning of pediatric proximal humeral fractures JBJS Essential Surg Tech 2019 9 04 e33.1 e33.6 10.2106/JBJS.ST.19.00002 PMC6974310 32051779

[5] Kim A E Chi H Niknam K Swarup I Management of Pediatric Proximal Humerus Fractures: Current Concept Review JPOSNA 2023 5 01 1 12. Available from:https://www.jposna.org/index.php/jposna/article/view/580 10.55275/JPOSNA-2023-580 PMC12088180 40433089

[6] Goldstein S Swarup I Noonan K J Percutaneous fixation of pediatric proximal humerus fractures: Master's surgical technique JPOSNA 2023 5 02 1 10. Available from:https://www.jposna.org/index.php/jposna/article/view/703 10.55275/JPOSNA-2023-703 PMC12088123 40433533

