# Supplementary material for: Kapandji-assisted Closed Reduction and Percutaneous K-wire Fixation for Proximal Humerus Fractures in Adolescents: A Technical Note
Source: Rev Bras Ortop (Sao Paulo). 2024 Aug 1;59(5):e789–92. doi: 10.1055/s-0044-1787549 (PMC11624935; doi:10.1055/s-0044-1787549)
Supplement: Related texts

# Research article: Redução fechada assistida por Kapandji e fixação percutânea com fio de Kirschner em fraturas do úmero proximal em adolescentes: Nota técnica

**Keywords:** adolescente, fio de Kirschner, fraturas do úmero, redução fechada

## Abstract

As fraturas proximais do úmero representam aproximadamente 3 a 5% de todas as fraturas pediátricas e em adolescentes, com maior incidência em crianças mais velhas, principalmente entre 10 e 15 anos de idade. Fraturas sem luxação ou com luxação mínima geralmente podem ser tratadas de forma conservadora. No entanto, o manejo das fraturas proximais do úmero com luxação ou instabilidade em adolescentes pode exigir intervenção cirúrgica; nesses casos, a redução fechada e a fixação percutânea (CRPP, na sigla em inglês) são as técnicas mais empregadas. A CRPP não é isenta de possíveis desafios e dificuldades potenciais. Este artigo tem como objetivo descrever uma técnica que pode facilitar a CRPP e minimizar as complicações associadas a esta abordagem cirúrgica.

## Introdução

As fraturas proximais do úmero são responsáveis por aproximadamente 3 a 5% de todas as fraturas pediátricas. A maior incidência é observada em crianças mais velhas, principalmente entre 10 e 15 anos de idade. 1 As crianças do sexo masculino tendem a ser mais acometidas do que as do sexo feminino. Essas fraturas são geralmente decorrentes de quedas, lesões esportivas ou traumatismos diretos na região do ombro.

O tratamento das fraturas proximais do úmero em adolescentes depende de vários fatores, incluindo características da fratura, idade e maturidade esquelética. Fraturas sem luxação ou com luxação mínima geralmente podem ser tratadas de forma conservadora com imobilização usando tipoias ou cintas. Entretanto, fraturas com luxação ou instabilidade em pacientes com potencial limitado de remodelamento podem necessitar de intervenção cirúrgica. A redução fechada e a fixação percutânea (CRPP) são técnicas cirúrgicas comumente empregadas para o tratamento de fraturas proximais do úmero com luxação ou instabilidade em adolescentes. 2 3 4 Embora esses procedimentos possam ser eficazes na obtenção do alinhamento anatômico e na promoção da consolidação da fratura, eles apresentam desafios e possíveis dificuldades. Este artigo tem como objetivo descrever uma técnica que pode facilitar a CRPP e minimizar as complicações associadas a esta abordagem cirúrgica.

## Descrição da técnica

A Tabela 1 descreve as indicações para o tratamento cirúrgico das fraturas proximais do úmero. A redução é considerada em fraturas com desvio superior a 33% (Neer III ou IV) ou angulação superior a 40 graus em pacientes até 2 anos antes de atingir a maturidade esquelética (meninos com idade ≥ 14 anos e meninas com idade ≥ 12 anos). 5 O paciente é posicionado em decúbito dorsal ou em cadeira de praia modificada para permitir a obtenção de imagens ortogonais ( Figs. 1 e 2 ). Subsequentemente, todo o membro superior é preparado com campo cirúrgico. Com o braço em adução, 2 fios de Kirschner de 2,0 mm são inseridos de forma percutânea e avançados até o sítio da fratura. Nesta posição, é mais fácil introduzir os pinos do que com o braço em abdução e rotação. É crucial evitar o nervo axilar durante a colocação percutânea dos pinos. O nervo está localizado a 6 cm da face anterolateral do acrômio e os pinos são colocados distalmente a esse ponto. Após a confirmação radiográfica da posição dos pinos, é realizada a redução fechada da fratura com tração, abdução e rotação. Caso a redução seja considerada inadequada, utiliza-se um pino de Steinmann de 2,5 mm para facilitar a redução (técnica de Kapandji). Ocasionalmente, o periósteo ou a cabeça longa do bíceps podem interferir no sítio da fratura, com necessidade de redução aberta por via deltopeitoral. Depois da redução, os pinos são avançados e o pino de Steinmann é removido. É importante evitar a penetração dos fios de Kirschner na superfície articular. A seguir, há realização de fluoroscopia contínua para observação da rotação interna e externa máxima e avaliação da estabilidade e da posição final dos pinos. Na presença de sinais de instabilidade da fratura, um terceiro pino cruzado de 2,0 mm é inserido. Os fios são então cortados, dobrados e mantidos fora da pele, permitindo sua retirada no consultório.

**Tabela 1:** Indicações para o tratamento cirúrgico de fraturas do úmero proximal em adolescentes

Indicações
Fraturas expostas.
Fraturas associadas a comprometimento neurovascular.
Elevação cutânea ou risco iminente de fratura exposta.
Crianças mais velhas, próximas à maturidade esquelética, com fraturas com luxações e anguladas.
Politraumatismo com necessidade de carga de peso precoce no membro superior acometido.
Fraturas ipsilaterais de membro (cotovelo flutuante).

**Fig. 1:** A) Imagem clínica e radiografias à apresentação inicial. B) Imagens fluoroscópicas durante a redução fechada e a fixação percutânea. C) Radiografias anteroposterior e em perfil aos 3 meses de acompanhamento.

**Fig. 2:** Diagrama mostrando a redução fechada assistida por Kapandji e a fixação percutânea com fio de Kirschner em fraturas proximais do úmero.

No período pós-operatório, o membro acometido é colocado em tipoia acolchoada para manter certo grau de abdução. A fratura é acompanhada quinzenalmente para avaliação da cicatrização e a tipoia e os pinos são removidos após 4 semanas no consultório. Exercícios passivos e progressivos ativos de amplitude de movimento do ombro, incluindo elevação para frente e rotação externa com o braço ao lado, bem como exercícios de pêndulo, começam depois da remoção do pino.

## Discussão

A fise proximal do úmero apresenta notável potencial de remodelação, com tolerância significativa a deformidades em crianças com esqueleto imaturo. No entanto, a intervenção cirúrgica pode ser necessária em determinadas situações. Este artigo apresenta uma técnica simples e eficaz que facilita a CRPP, minimizando o tempo operatório e as complicações associadas a esta abordagem cirúrgica ( Tabela 2 ).

**Tabela 2:** Recomendações para evitar os desafios comuns na redução fechada e colocação percutânea de pinos em fraturas do úmero proximal em adolescentes

Desafios	Como evitar
Lesão do nervo axilar	O nervo está localizado a 6 cm da face anterolateral do acrômio e os pinos devem ser colocados distalmente a esse ponto.
Penetração da superfície articular	Realização de fluoroscopia contínua para avaliação da posição final dos pinos em todas as projeções.
Perda de fixação	Realização de fluoroscopia contínua em rotação interna e externa máxima para avaliação da estabilidade.
Necrose cutânea	Avaliação da necessidade de incisões de relaxamento cutâneo no sítio de inserção do pino.
Infecção no sítio do pino	Administração de antibióticos profiláticos, cuidados com pinos e observação atenta

A utilização da CRPP é associada a dois desafios principais: 1) a redução, pois o controle do fragmento proximal é bastante difícil devido às forças musculares e 2) a fixação, uma vez que alcançar o ângulo de entrada oblíquo necessário para que os pinos atravessem a fratura pode representar dificuldades.

A manobra de redução fechada requer a aplicação de tração axial, abdução e rotação externa do braço. No entanto, a inserção de fios de Kirschner nessa posição apresenta desafios significativos devido ao ângulo de entrada oblíquo necessário para atravessar a fratura no osso cortical duro. Para superar essa dificuldade, foi proposto o uso de uma guia de perfuração para garantir uma ancoragem mais segura do fio. No entanto, é comum que os pinos deslizem ao tentar uma trajetória distal-proximal. 6 Com base em nossa experiência, constatamos que a colocação percutânea de dois fios de Kirschner de 2,0 mm até atingir a fratura com o braço em adução é mais fácil do que a introdução dos pinos na posição de redução. Uma vez confirmada a posição adequada dos pinos à fluoroscopia, é realizada uma redução fechada da fratura e os pinos são avançados na epífise. Tradicionalmente, se essa manobra não obtivesse redução satisfatória, seria realizada uma redução aberta com abordagem deltopeitoral, o que não é isento de riscos. Na técnica proposta, nas situações em que a redução é inadequada em manobras fechadas, utiliza-se um pino de Steinmann de 2,5 mm para auxiliar a redução com uma manobra de tipo Kapanji. Essa técnica pode auxiliar a obtenção do alinhamento adequado da fratura, evitando a redução aberta. Recentemente, Goldstein et al. 6 propuseram o uso de uma pinça hemostática romba para fins semelhantes. Esse instrumento permite o acesso seguro ao sítio da fratura, alavanca o córtex do fragmento distal e o translada para alinhamento com o úmero proximal.

## Considerações finais

Em resumo, a maioria das fraturas proximais do úmero na população pediátrica e adolescente pode ser tratada sem cirurgia devido ao seu notável potencial de remodelação. Contudo, nos casos em que a angulação e o deslocamento excedem os limites aceitáveis, a nossa técnica de CRPP pode ser uma abordagem fiável para alcançar uma redução satisfatória e uma fixação estável. Este método é um meio reprodutível para tratamento de fraturas que exigem intervenção cirúrgica.
